# Supplementary material for: Extrinsically microporous polymer membranes derived from thermally cross-linked perfluorinated aryl-ether-free polymers for gas separation
Source: Nat Commun. 2025 Aug 5;16:7143. doi: 10.1038/s41467-025-62372-y (PMC12325600; doi:10.1038/s41467-025-62372-y)
Supplement: Supplementary file 1 — Supplementary Information [file 41467_2025_62372_MOESM1_ESM.pdf]

Supplementary Information

Extrinsically Microporous Polymer Membranes Derived from Thermally Cross-linked Perfluorinated Aryl-Ether-Free Polymers for Gas Separation

Ju Ho Shin[1], Hyun Jung Yu[1], Jiyoon Jung[2], Heseong An[3], Jung Hoon Park[4], Albert S. Lee[2,5,*], and Jong Suk Lee[1,6,7,*]

[1] Department of Chemical and Biomolecular Engineering, Sogang University, 35 Baekbeom-ro, Mapo-gu, Seoul 04107, Republic of Korea.

[2] Materials Architecture Research Center, Korea Institute of Science and Technology, Hwarang-ro 14-gil 5, Seongbuk-gu, Seoul, 02792, Republic of Korea.

[3] Department of Chemical Engineering, Sunchon National University, 255 Jungang-ro, Suncheon-si, Jeollanam-do, 57922, Republic of Korea

[4] Department of Chemical and Biochemical Engineering, Dongguk University, 30 Pildong-ro 1gil, Jung-gu, Seoul, 04620, Republic of Korea

[5] Convergence Research Center for Solutions to Electromagnetic Interference in Future-Mobility, Korea Institute of Science and Technology (KIST), Seoul 02792, Republic of Korea.

[6] Institute of Energy and Environmental Technology, Sogang University, 35 Baekbeom-ro, Mapo-gu, Seoul 04107, Republic of Korea

[7] Institute of Emergent Materials, Sogang University, 35 Baekbeom-ro, Mapo-gu, Seoul 04107, Republic of Korea

*Correspondence to: jongslee@sogang.ac.kr; aslee@kist.re.kr

Table of Contents

Supplementary Note 1. Detailed supplementary characterizations. 3

Supplementary Note 2. Preparation of the CMS membranes. 4

Supplementary Note 3. Preparation of asymmetric *p*TPPFA EMP hollow fiber membranes........ 4

Supplementary Note 4. Calculation of dual-mode transport model. 5

Supplementary Note 5. Single-gas permeation experiments at sub-ambient temperature. 5

Supplementary Fig. 1. Characterizations of polymers (a) Synthesis of *p*TPTFA and *p*TPPFA. ^{1}H NMR (400 MHz, $CDCl_3$) spectra of (b) *p*TPTFA and (c) *p*TPPFA. 6

Supplementary Fig. 2. The hypothetical cavity morphologies of a glassy polymer, an extrinsically microporous polymer, and a carbon molecular sieve (CMS) membrane. 7

Supplementary Fig. 3. MS spectra of compounds evolved during pyrolysis of *p*TPPFA at (a) 400 °C, (b) 450 °C, and (c) 500 °C. 8

Supplementary Fig. 4. MS spectra of compounds evolved during pyrolysis of *p*TPPFA at (a) 550 °C and (b) 600 °C. 9

Supplementary Fig. 5. MS spectra of compounds evolved during pyrolysis of *p*TPTFA at (a) 400 °C, (b) 450 °C, and (c) 500 °C. 10

Supplementary Fig. 6. MS spectra of compounds evolved during pyrolysis of *p*TPTFA at (a) 550 °C and (b) 600 °C. 11

Supplementary Fig. 7. Selected ion monitoring using TG-GC/MS of compounds evolved during the pyrolysis of *p*TPTFA. 12

Supplementary Fig. 8. DSC plots of *p*TPPFA and thermally treated *p*TPPFA membranes. 13

Supplementary Fig. 9. Modulated DSC plots of *p*TPPFA. 14

Supplementary Fig. 10. Modulated DSC plots of thermally treated *p*TPPFA at 400 °C. 15

Supplementary Fig. 11. Modulated DSC plots of thermally treated *p*TPPFA at 450 °C. 16

Supplementary Fig. 12. Modulated DSC plots of thermally treated *p*TPPFA at 500 °C. 17

Supplementary Fig. 13. TGA plots of *p*TPPFA films subjected to different thermal treatment protocols under argon purge, with an Ar purge rate of 50 ml min^{-1}. 18

Supplementary Fig. 14. Hardness and reduced modulus of *p*TPPFA and thermally treated membranes measured by nanoindentation at 25 °C. Error bars represent standard deviations for five measurements. 19

Supplementary Fig. 15. Stress-strain curves of *p*TPPFA and *p*TPPFA 450 °C. 20

Supplementary Fig. 16. (a) XPS survey spectra, (b) high-resolution C 1*s* XPS spectra, and (c) F 1*s* XPS spectra of *p*TPPFA and thermally treated membranes. Deconvolution of the C1s XPS spectra of (d) *p*TPPFA, (e) *p*TPPFA 400 °C, and (f) *p*TPPFA 450 °C. 21

Supplementary Fig. 17. Digital photos of thermally treated *p*TPPFA membranes immersed in NMP. 22

Supplementary Fig. 18. CO_2 adsorption isotherms of *p*TPPFA and thermally treated *p*TPPFA membranes at 0 °C. 23

Supplementary Fig. 19. Raman spectra of the *p*TPTFA 500 °C. The yellow line represents the raw data, which show only readout noise. 24

Supplementary Fig. 20. Wide-angle X-ray diffraction patterns of *p*TPTFA and *p*TPTFA 500 °C. The characteristic peak for the aluminum sample holder is highlighted with an asterisk. 25

Supplementary Fig. 21. A proposed chemical structure of *p*TPTFA 500 °C. 26

Supplementary Fig. 22. Diffusivity and solubility selectivity of CO_2/N_2 and CO_2/CH_4 for *p*TPPFA and its thermally treated derivatives at 1 bar and 35 °C. 27

Supplementary Fig. 23. Comparisons of the single-gas separation performance of thermally treated *p*TPPFA for (a) C_2H_4/C_2H_6 and (b) C_3H_6/C_3H_8 measured at 2 bar and 35 °C. 28

Supplementary Fig. 24. Long-term mixed-gas permeation performance of *p*TPPFA 450 °C over 96 hours of continuous operation. 29

Supplementary Fig. 25. Equimolar CO_2/CH_4 mixed-gas separation performance as a function of total feed pressure for *p*TPPFA at 35 °C. 30

Supplementary Fig. 26. Photographs of (a) the exterior and (b) the interior of the in-house permeation system for sub-ambient temperature. 31

Supplementary Table 1. Molecular weight, glass transition temperature, density, fractional free volume, and *d*-spacing of *p*TPTFA and *p*TPPFA. 32

Supplementary Table 2. Summary of mechanical properties of *p*TPPFA and *p*TPPFA 450°C membranes obtained from tensile stress-strain measurements. 32

Supplementary Table 3. BET surface area of EMPMs derived from thermally cross-linked *p*TPPFA and *p*TPTFA. 32

Supplementary Table 4. Dual-mode parameters of *p*TPPFA and EMPM (450 °C) for CO_2 adsorption isotherms. 33

Supplementary Table 5. Partial immobilization model diffusion parameters for CO_2 adsorption isotherms. 33

Supplementary Table 6. Single-gas permeation results of freshly prepared asymmetric *p*TPPFA EMP hollow fiber membranes at 1 bar. 33

Supplementary Table 7. Long-term single-gas permeation results of asymmetric *p*TPPFA EMP hollow fiber membranes at 1 bar and sub-ambient temperature (-20 °C). The testing module was stored under a vacuum at -20 °C. 34

Supplementary Table 8. Mixed-gas permeation results of asymmetric *p*TPPFA EMP hollow fiber membranes at 2 bar and sub-ambient temperature (-20 °C). 34

Supplementary Table 9. Comparison of CO_2/N_2, CO_2/CH_4 separation performance between polymeric membranes and thermally cross-linked membranes. 35

Supplementary Note 1. Detailed supplementary characterizations.

^1H nuclear magnetic resonance (NMR) spectra were collected using a Bruker Avance III HD 400 MHz spectrometer. The weight-averaged molecular weight (M_w) and molecular weight distributions (M_w/M_n) of the polymers were measured with a JASCO PU-2080 plus SEC system equipped with a refractive index detector (RI-2031 plus) and a UV detector (λ = 254 nm, UV-2075 plus), using THF as the mobile phase at 40 °C with a flow rate of 1 ml min^{-1}. Samples were separated through four columns (Shodex-GPC KF-802, KF-803, KF-804, KF-805). The bulk density of the polymers was determined with a helium pycnometer (AccuPyc 1340, Micrometrics) at 25 ^oC from 10 cycles. Before measurement, each sample was activated under vacuum for at least 12 h. The glass transition temperatures of the polymer and thermally treated polymer were measured using DSC (DSC25, Discovery) with a ramp rate of 10 ^oC min^{-1} under nitrogen. Modulated DSC (mDSC) was conducted to evaluate the kinetic component (non-reversing heat flow) during thermal treatment, related to polymer carbonization. Temperature modulation was 1 ^oC for 60 s, with a ramp rate of 2 ^oC min^{-1} under nitrogen. Fractional free volumes of *p*TPTFA and *p*TPPFA were calculated from the specific volume measured at 25 ^oC and an estimated occupied volume based on Bondi's group contribution method[1]. Evolved gases during heat treatment were analyzed using an STA 449 F5 Jupiter (Netzsch) coupled with an Agilent GC/MS system with a heating rate of 10 ^oC min^{-1} under a nitrogen atmosphere. Actual weight losses of polymer films were measured with a Discovery SDT-650 (TA Instruments-Waters LLC) following the exact heat treatment protocol used for thermally treated films. Nanoindentation tests were conducted using a Hysitron TI-950 TriboIndenter (Bruker) with a Berkovich indenter tip at a maximum load of 5 mN. Reduced modulus and hardness values were averaged from five tests per sample. Hardness is defined as the resistance of a sample to deformation by the indenter tip. Tensile tests were carried out using a Shimadzu Autograph AGS-X precision universal testing machine equipped with a 500 N load cell. Membranes with thicknesses ranging from 30 to 50 μm were cut into regular specimens with approximately 20 mm in an effective length and approximately 8 mm in width. Young's modulus was determined from the initial linear region of the stress-strain curve. Tensile strength at break and elongation at break were obtained by averaging the results from three independent tests performed on separate membrane specimens. X-ray photoelectron spectroscopy (XPS) was performed on a Nexsa instrument (Thermo Fisher Scientific) with a monochromatic aluminum Kα (1486.6 eV) X-ray source.

Supplementary Note 2. Preparation of the CMS membranes.

*p*TPPFA films underwent thermal treatment at 550 °C in a three-zone furnace from Thermocraft. The procedure involved heating the membranes from 50 °C to 250 °C at 13.3 °C min^{-1}, followed by further heating to 535 °C at 3.85 °C min^{-1}, and finally to 550 °C at 0.25 °C min^{-1}. Subsequently, the membranes were allowed to soak at 550 °C for 2 h, after which the furnace was allowed to cool naturally under an argon purge.

Supplementary Note 3. Preparation of asymmetric *p*TPPFA EMP hollow fiber membranes.

The α-Al_2O_3 supports were attached to a dip coater (RCD-15, Bungard) and immersed in a boehmite alumina solution at a rate of 1 cm s^{-1}. The supports were soaked in the solution for 30 s and taken out at a withdrawal rate of 0.1 cm s^{-1}. To prevent coating the internal surface, one end of each hollow fiber substrate was sealed with epoxy (3 M™ scotch-weld™) prior to dip coating. The γ-Al_2O_3-coated supports were then dried vertically in the atmosphere for 24 h and transferred to a furnace for sintering at 500 °C for 4 h, with a heating rate of 4 °C min^{-1}. The γ-Al_2O_3 coated α-Al_2O_3 hollow fiber supports were coated with dope solution (7 wt%, THF) using a dip coating at room temperature. One end of the fibers was sealed with epoxy and then attached to the dip coater. Then, they were dipped into the dope solution at an immersion rate of 1 cm s^{-1}, a withdrawing rate of 0.1 cm s^{-1}, and a 30 s immersion time. The fabricated asymmetric *p*TPPFA hollow fiber membranes were dried vertically in the atmosphere at 28 °C for 10 min and subsequently transferred to a vacuum oven for secondary drying at 80 °C for 12 h under vacuum to remove any residual solvents. Lastly, they were converted into EMPM at 450 °C for 2 h in a three-zone split-tube furnace, following the same procedure used for the fabrication of thermally treated membranes.

Supplementary Note 4. Calculation of dual-mode transport model.

The CO_2 permeability was modeled using a partial immobilization model with dual-mode parameters as described by the equation below[2]:

$$P_{CO_2} = k_{D,CO_2} \cdot D_{D,CO_2} + \frac{C'_{H,CO_2} b_{CO_2} \cdot D_{H,CO_2}}{1 + b_{CO_2} \cdot p}$$

Alternatively,

$$P_{CO_2} = k_{D,CO_2} \cdot D_{D,CO_2} + \frac{F_{CO_2} \cdot K_{CO_2}}{1 + b_{CO2} \cdot p}$$

Where $F_{CO_2} = \frac{D_{H,CO_2}}{D_{D,CO_2}}$, $K_{CO_2} = \frac{C'_{H,CO_2} b_{CO_2}}{k_{D,CO_2}}$, and p represents the feed gas pressure.

The parameters D_{D,CO_2} and F_{CO_2} were determined by non-linear regression of the permeability data as a function of feed pressure for *p*TPPFA and EMPM.

Supplementary Note 5. Single-gas permeation experiments at sub-ambient temperature.

The permeation cell was enclosed in a climate chamber (SH-CH-150U1, Samheung Energy) allowing precise temperature control over the range of -20 to 35 °C, with an accuracy of ±0.1 °C. Prior to each measurement, the permeation cell was degassed for 24 h. Following degassing, the leak rate was measured by isolating the cell from the vacuum pump. The permeation system's leak rate was below 1×10^{-6} torr s^{-1} with a downstream volume of 96.5 cm^3 at -20 °C.

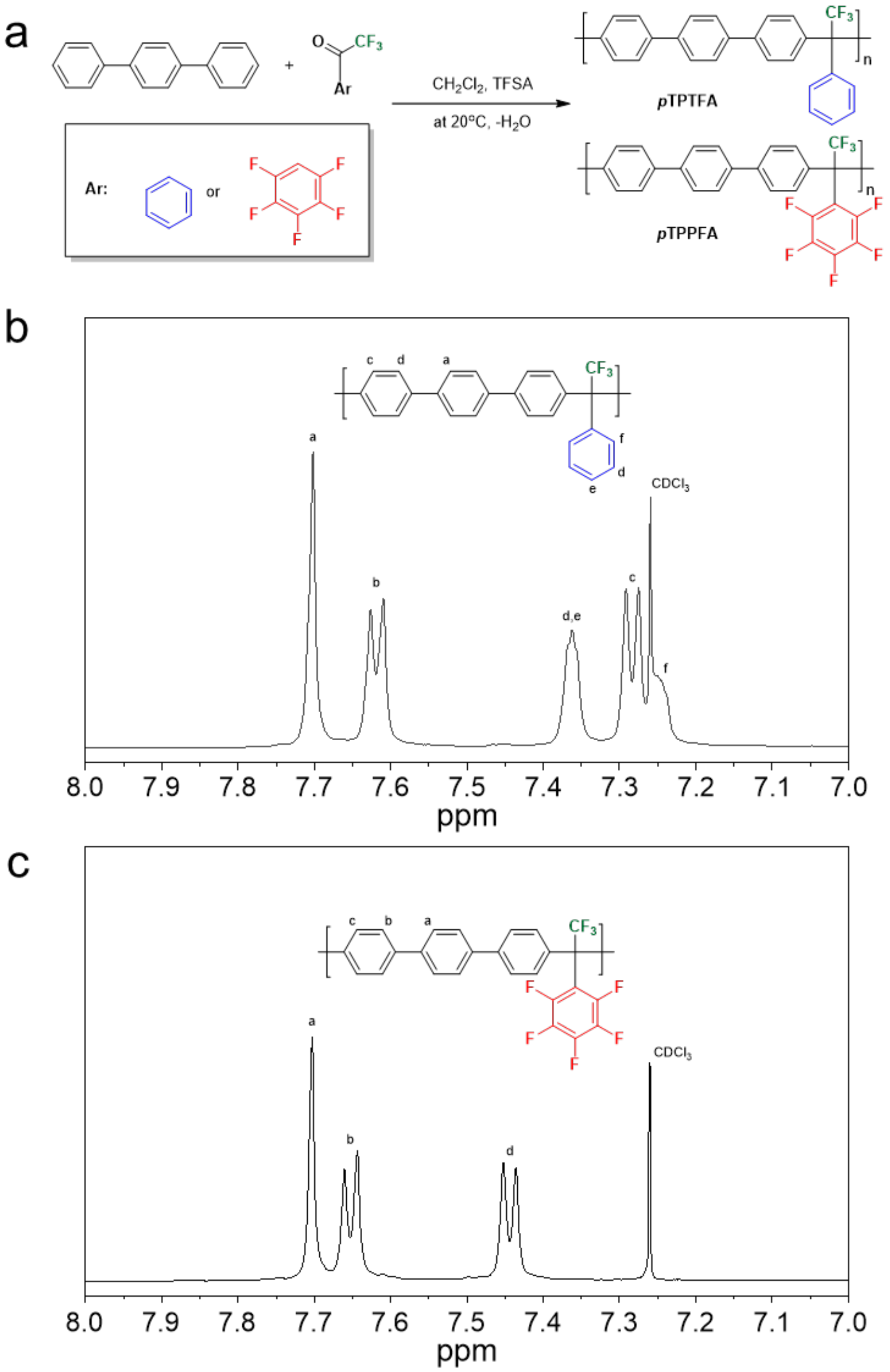

Supplementary Fig. 1. Characterizations of polymers (a) Synthesis of *p*TPTFA and *p*TPPFA. ^{1}H NMR (400 MHz, $CDCl_3$) spectra of (b) *p*TPTFA and (c) *p*TPPFA.

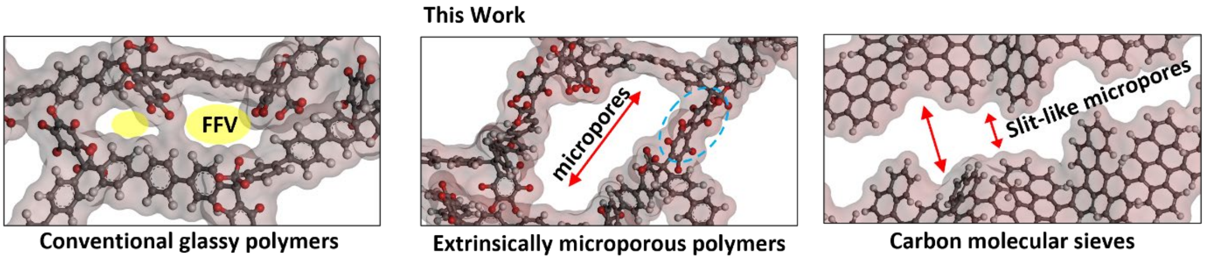

Supplementary Fig. 2. The hypothetical cavity morphologies of a glassy polymer, an extrinsically microporous polymer, and a carbon molecular sieve (CMS) membrane.

The Connolly surface of the hypothetical polymer strands is shown in shaded red regions (outer surface), geometrically outlined by an N_2-sized probe (1.45Å). Conventional glassy polymers possess excess free volume, originating from random thermal fluctuations of polymer chains, which facilitates gas transport through the polymer. In extrinsically microporous polymers, inter-chain cross-linking induced by defluorination creates a microporous structure. CMS membranes, formed through controlled high-temperature pyrolysis of polymeric membranes, exhibit well-defined bimodal pore structures.

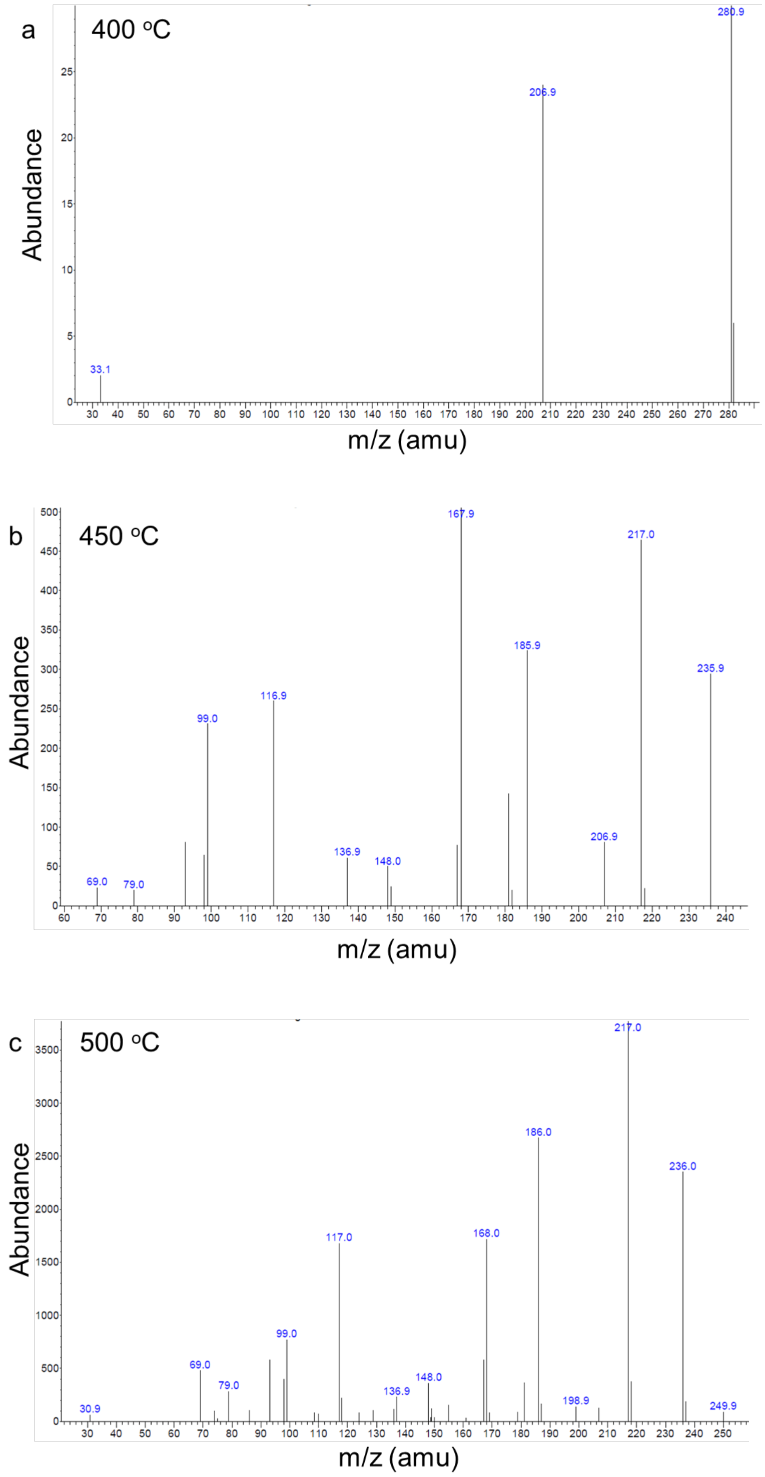

Supplementary Fig. 3. MS spectra of compounds evolved during pyrolysis of *p*TPPFA at (a) 400 °C, (b) 450 °C, and (c) 500 °C.

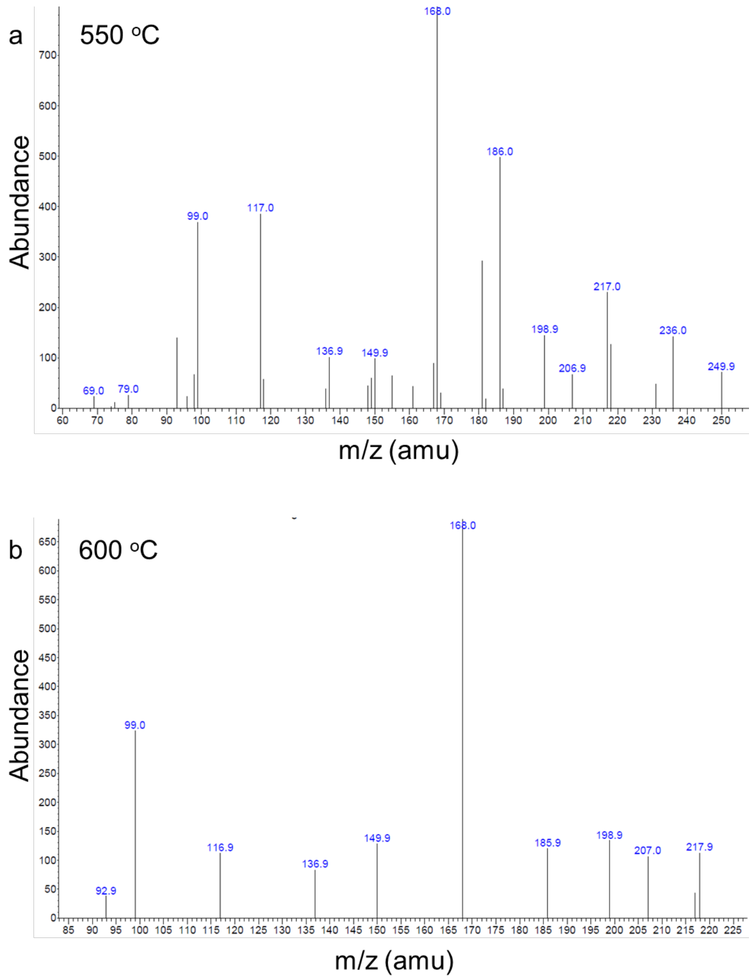

Supplementary Fig. 4. MS spectra of compounds evolved during pyrolysis of *p*TPPFA at (a) 550 °C and (b) 600 °C.

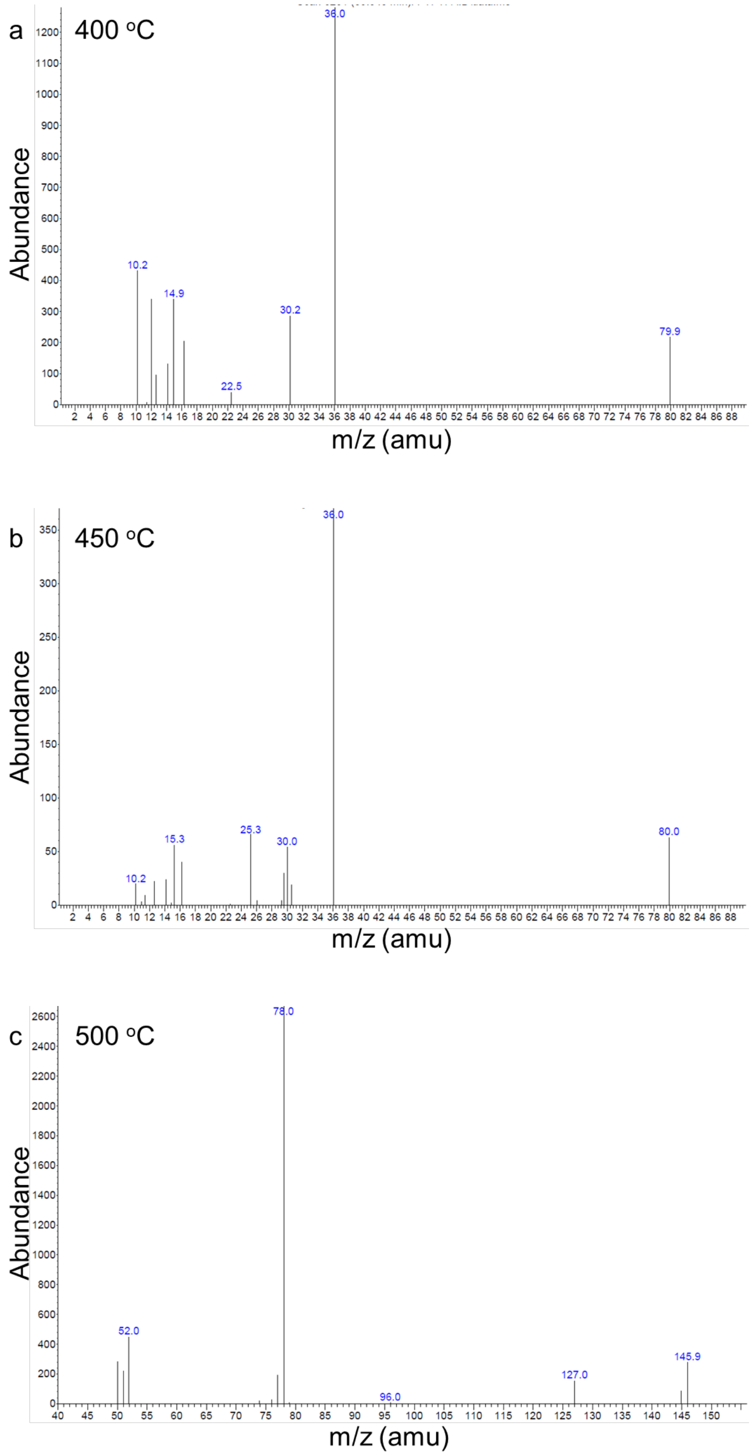

Supplementary Fig. 5. MS spectra of compounds evolved during pyrolysis of *p*TPTFA at (a) 400 °C, (b) 450 °C, and (c) 500 °C.

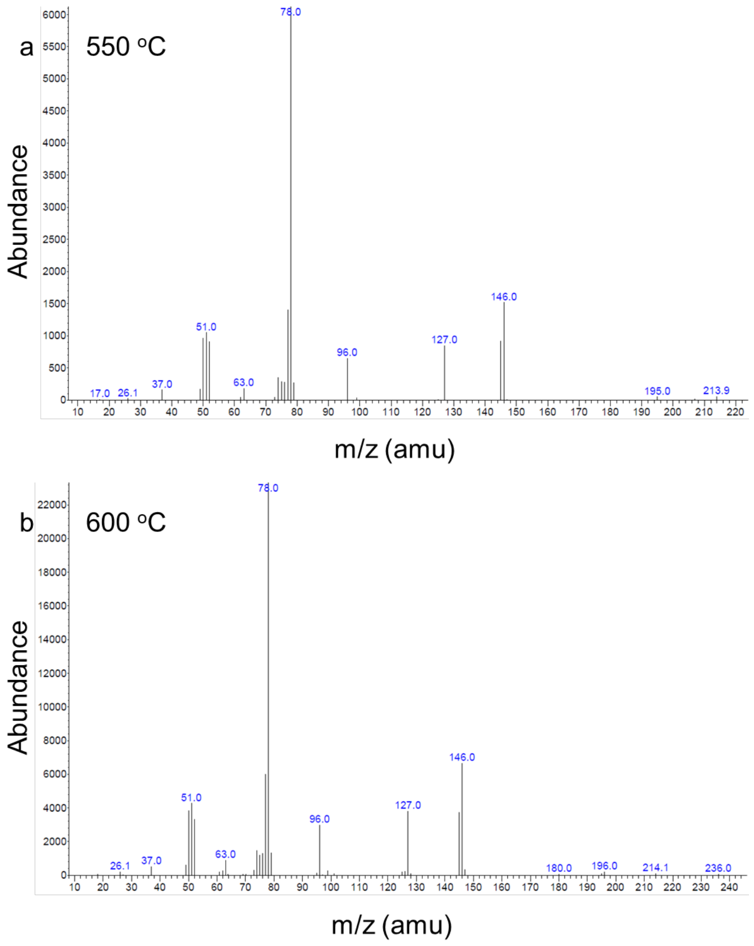

Supplementary Fig. 6. MS spectra of compounds evolved during pyrolysis of *p*TPTFA at (a) 550 °C and (b) 600 °C.

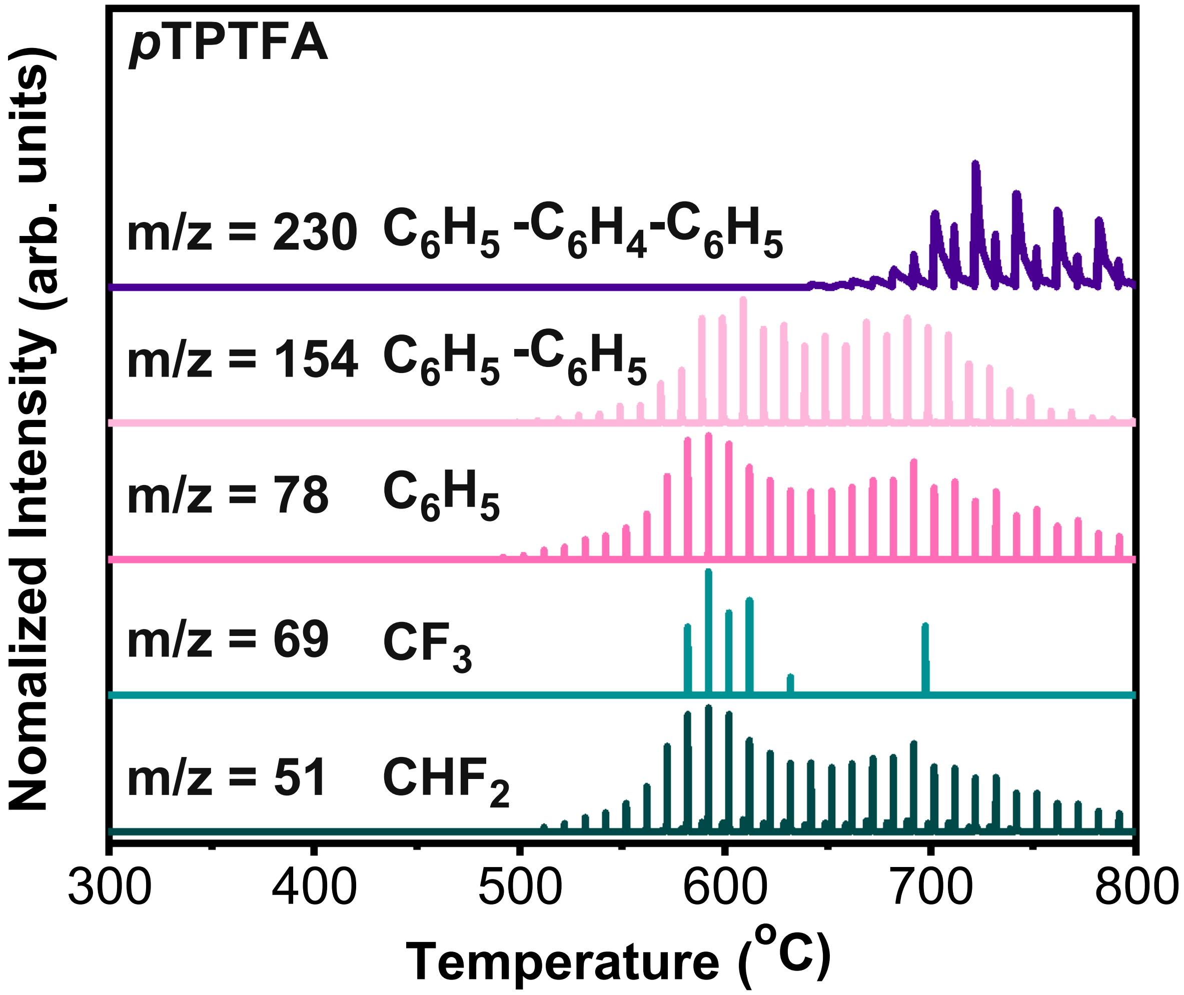

Supplementary Fig. 7. Selected ion monitoring using TG-GC/MS of compounds evolved during the pyrolysis of *p*TPTFA.

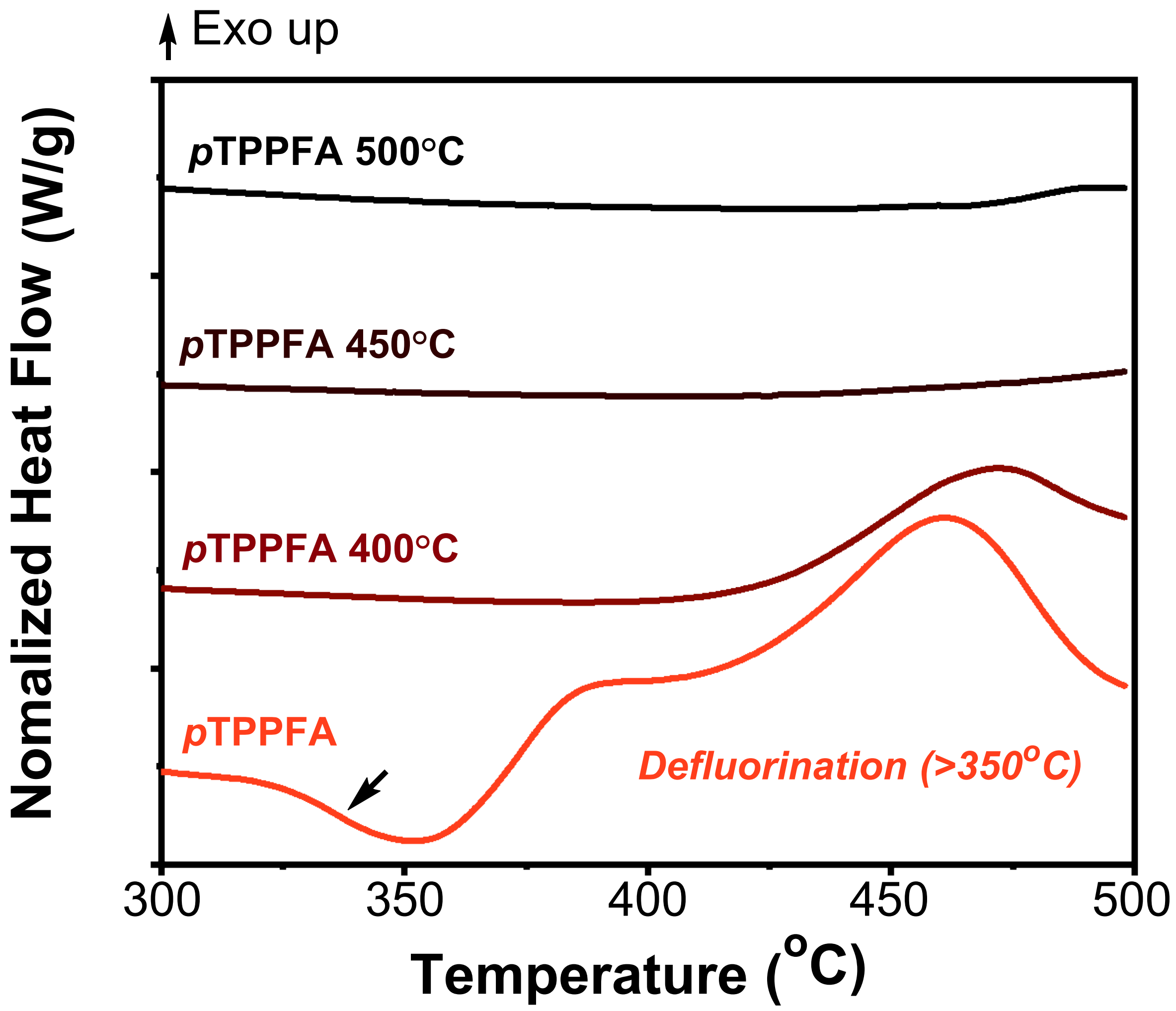

Supplementary Fig. 8. DSC plots of *p*TPPFA and thermally treated *p*TPPFA membranes.

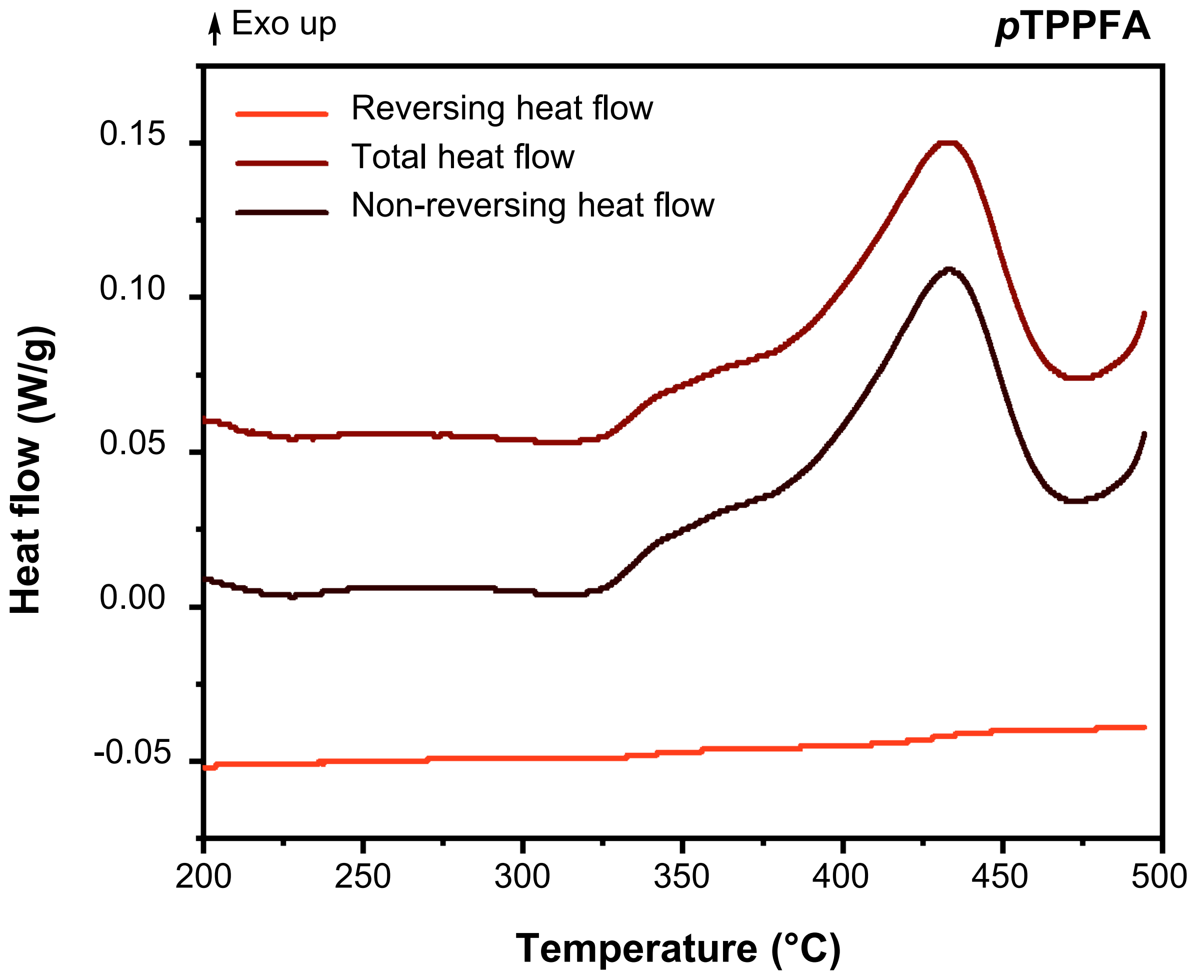

Supplementary Fig. 9. Modulated DSC plots of *p*TPPFA.

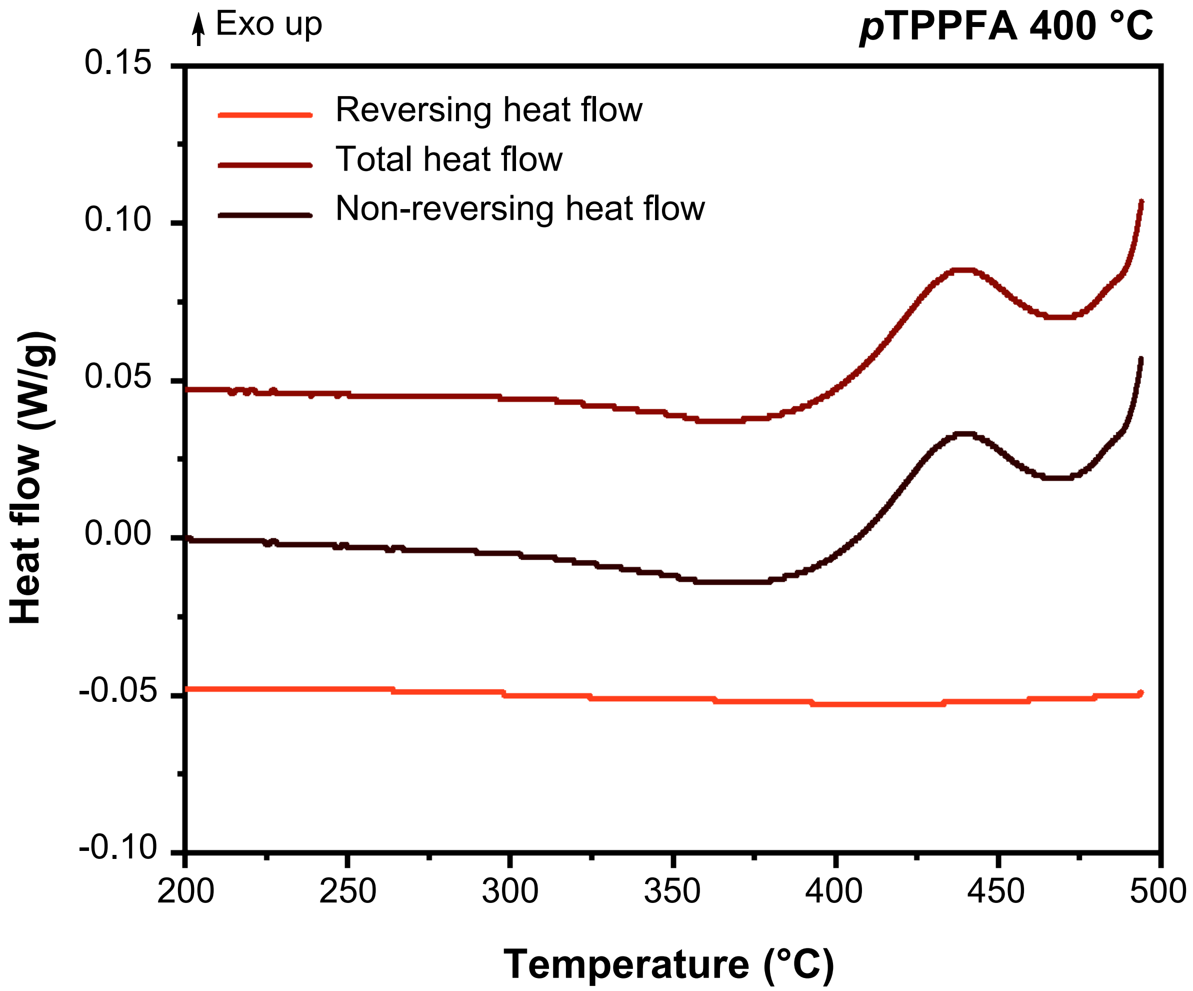

Supplementary Fig. 10. Modulated DSC plots of thermally treated *p*TPPFA at 400 °C.

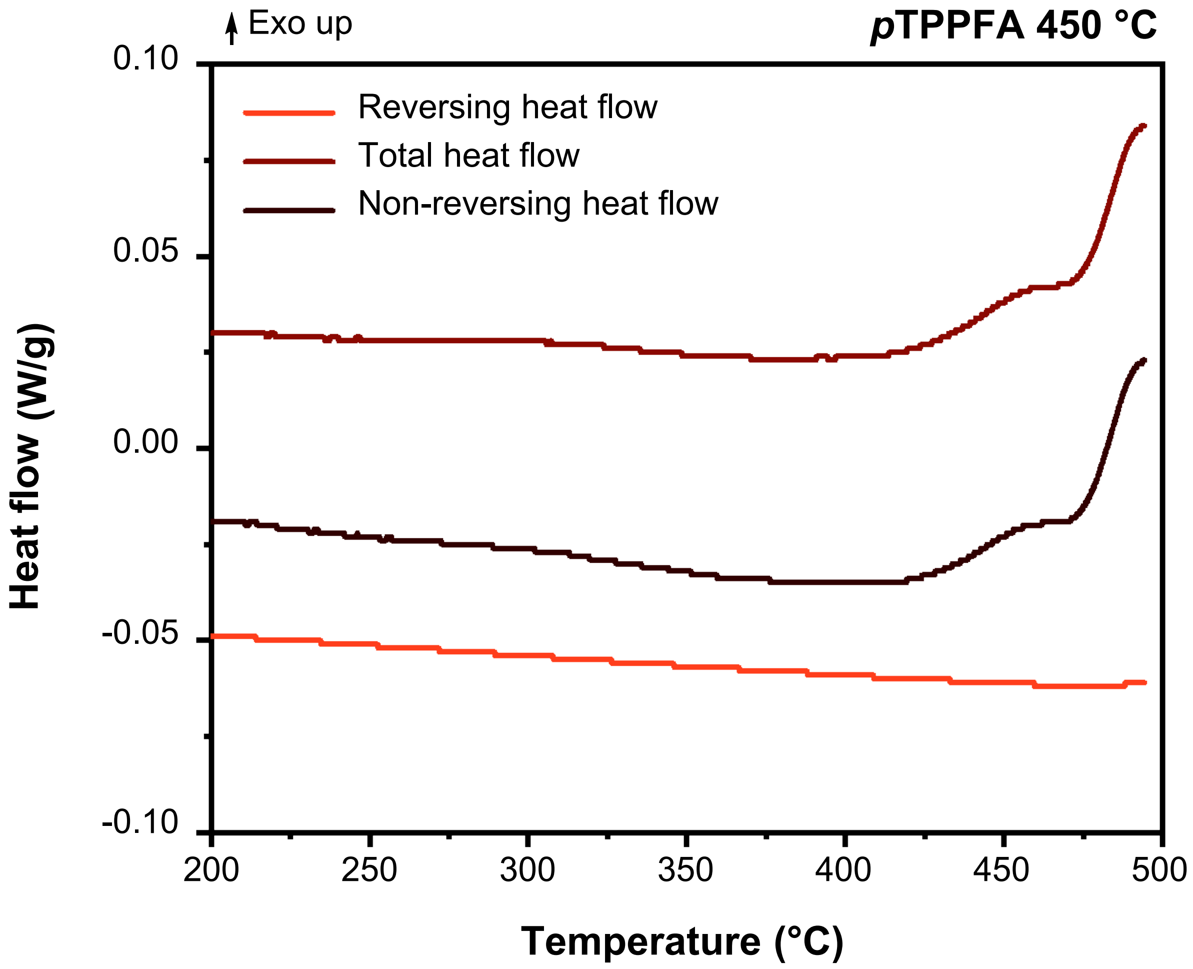

Supplementary Fig. 11. Modulated DSC plots of thermally treated *p*TPPFA at 450 °C.

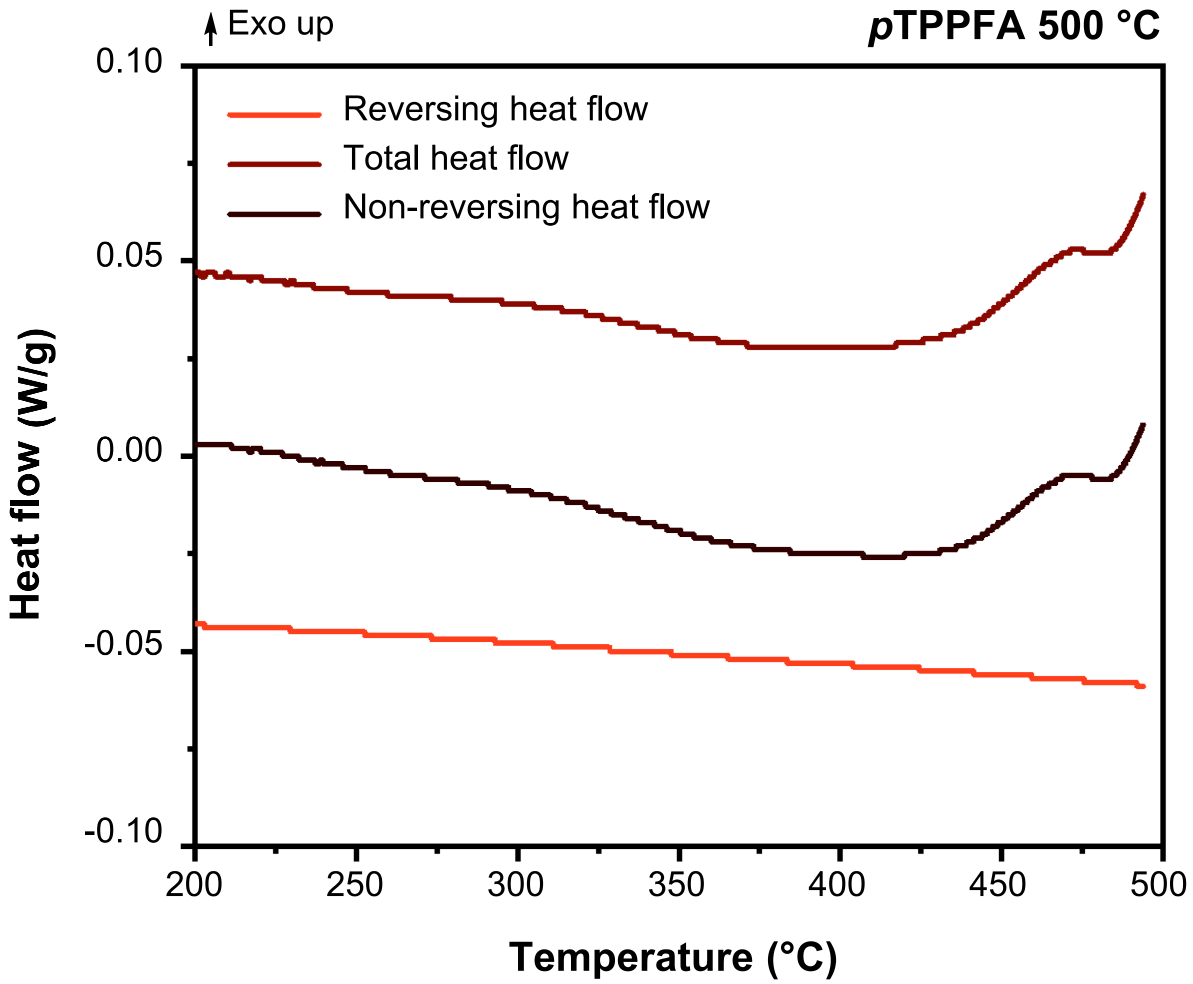

Supplementary Fig. 12. Modulated DSC plots of thermally treated *p*TPPFA at 500 °C.

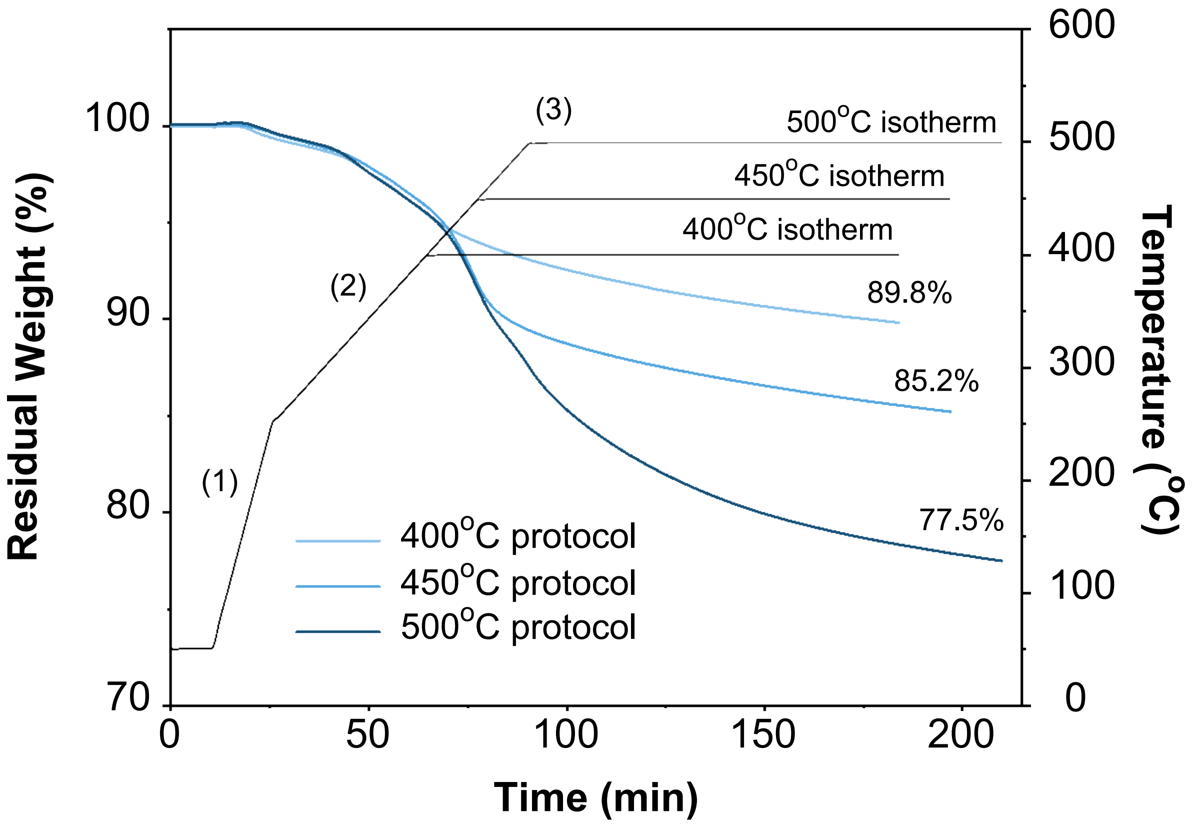

Supplementary Fig. 13. TGA plots of *p*TPPFA films subjected to different thermal treatment protocols under argon purge, with an Ar purge rate of 50 ml min^{-1}.

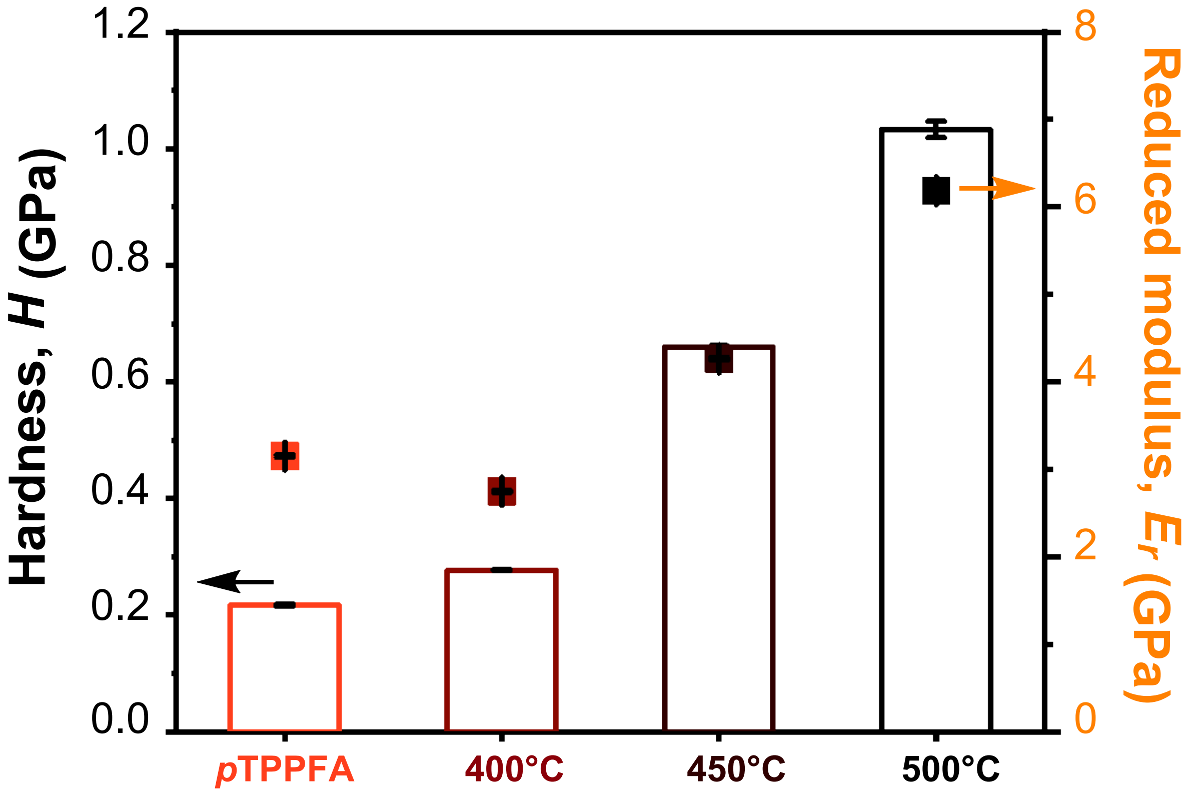

Supplementary Fig. 14. Hardness and reduced modulus of *p*TPPFA and thermally treated membranes measured by nanoindentation at 25 °C. Error bars represent standard deviations for five measurements.

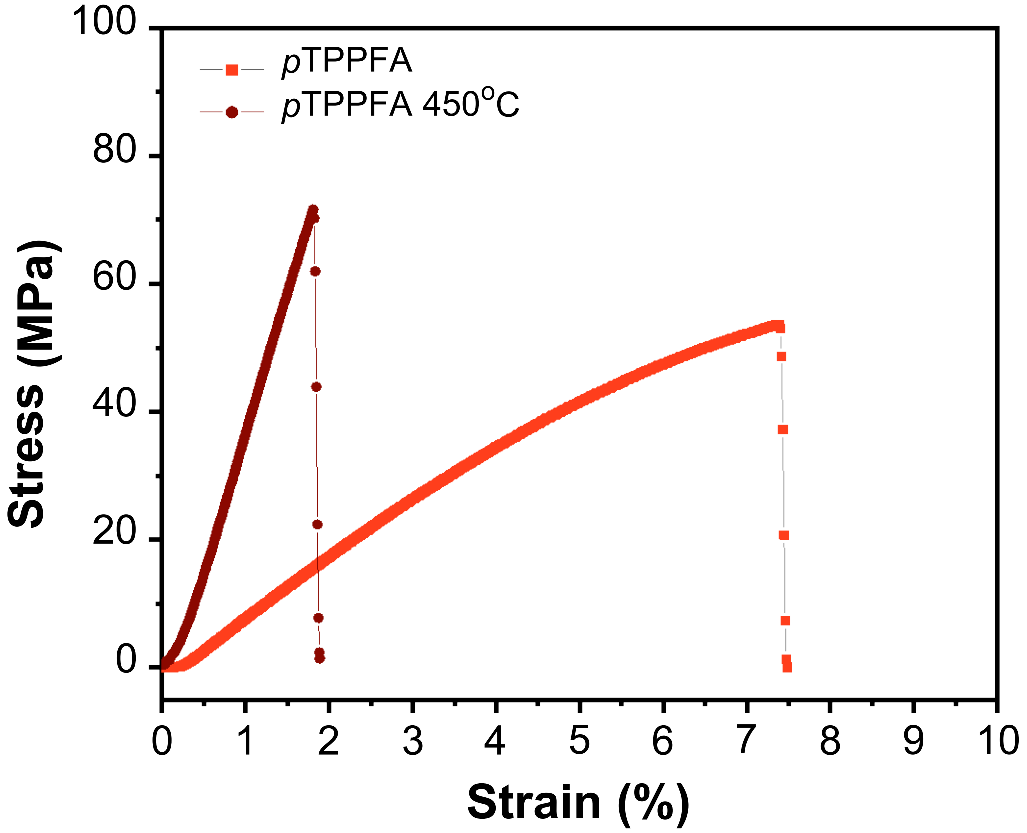

Supplementary Fig. 15. Stress-strain curves of *p*TPPFA and *p*TPPFA 450 °C.

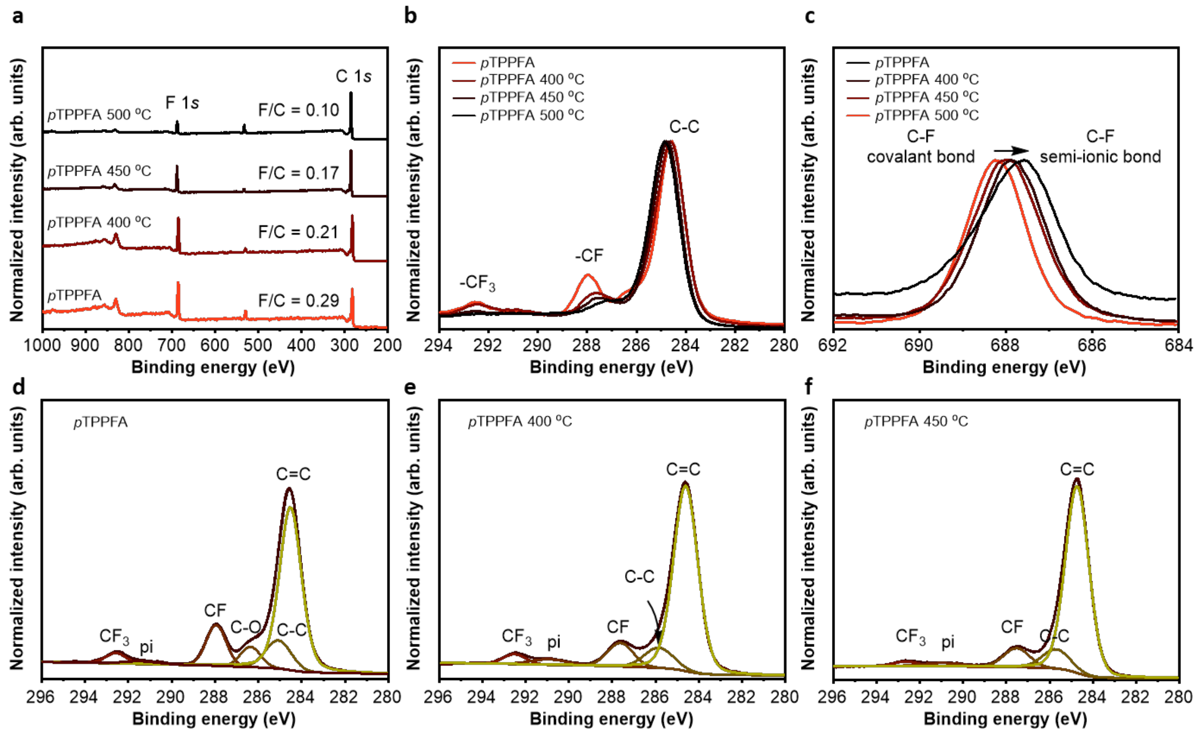

Supplementary Fig. 16. (a) XPS survey spectra, (b) high-resolution C 1*s* XPS spectra, and (c) F 1*s* XPS spectra of *p*TPPFA and thermally treated membranes. Deconvolution of the C1s XPS spectra of (d) *p*TPPFA, (e) *p*TPPFA 400 °C, and (f) *p*TPPFA 450 °C. XPS spectra were analyzed using XPSpeak41 software, employing Gaussian-Lorentzian lines for peak fitting. Prior to fitting, a combination of Shirley-linear background correction was applied to remove the spectrum background. The C 1*s* spectrum was used as a reference, with the adventitious carbon peak set to 284.6 eV. Five distinct peaks were fitted at C=C (284.7 eV), C-C (285.4 eV), C-F (287.6 eV), π-π* shake-up (291 eV), and -CF_3 (292.4 eV). An additional peak at C-O (286.6 eV) was fitted for *p*TPPFA, attributed to surface impurities.

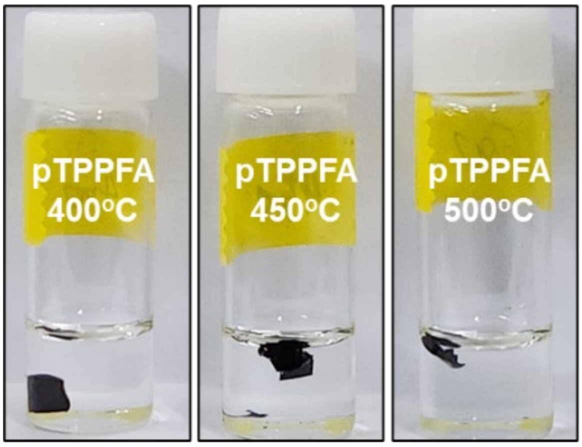

Supplementary Fig. 17. Digital photos of thermally treated *p*TPPFA membranes immersed in NMP.

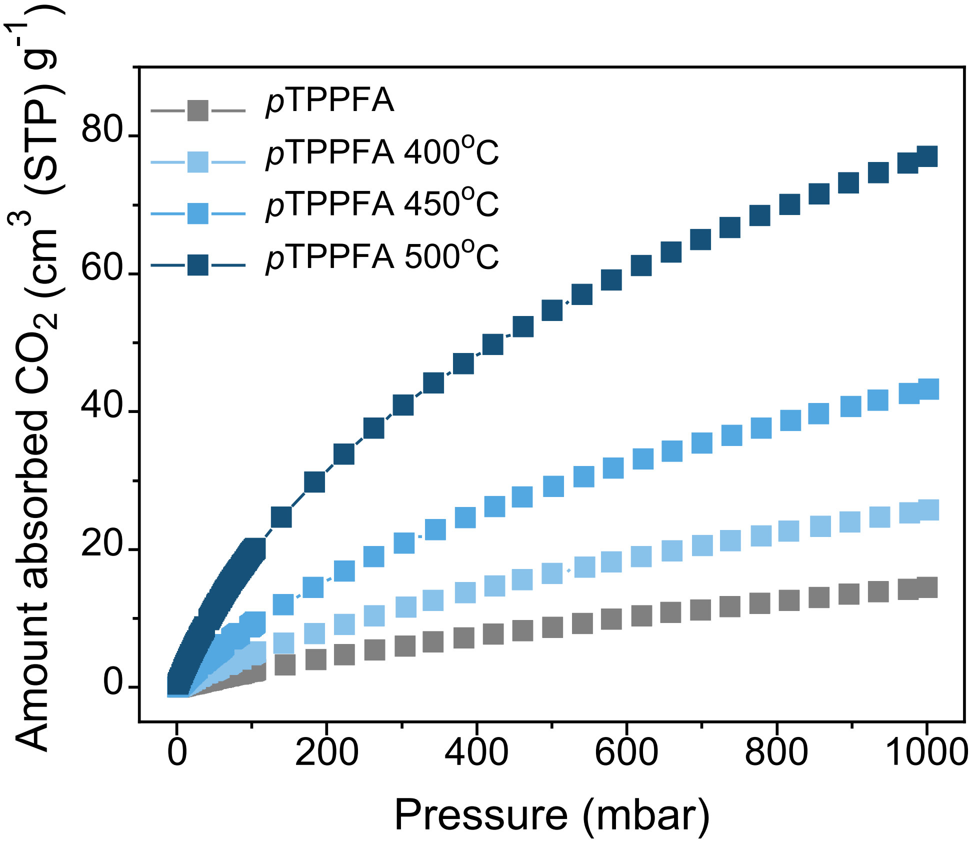

Supplementary Fig. 18. CO_2 adsorption isotherms of *p*TPPFA and thermally treated *p*TPPFA membranes at 0 °C.

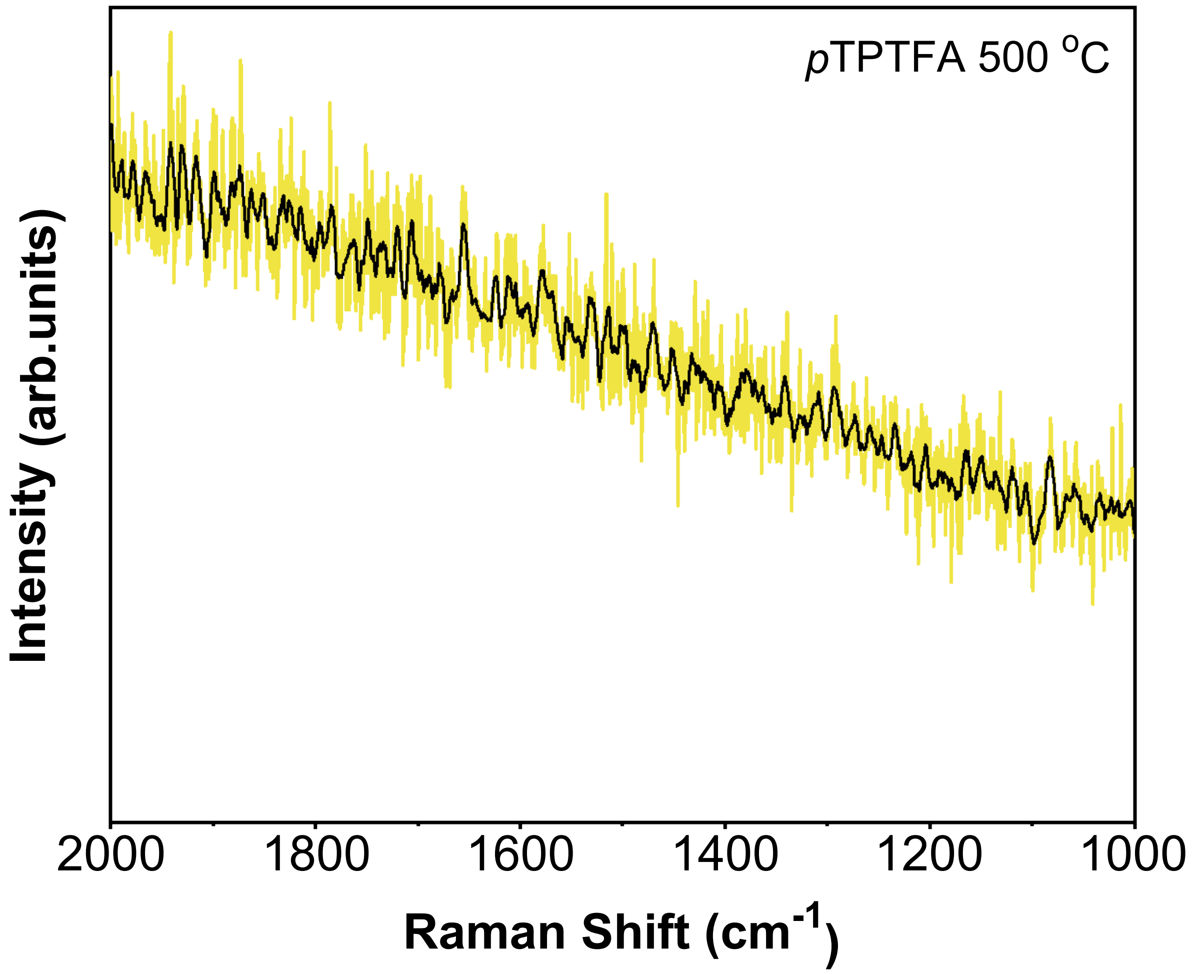

Supplementary Fig. 19. Raman spectra of the *p*TPTFA 500 °C**.** The yellow line represents the raw data, which show only readout noise.

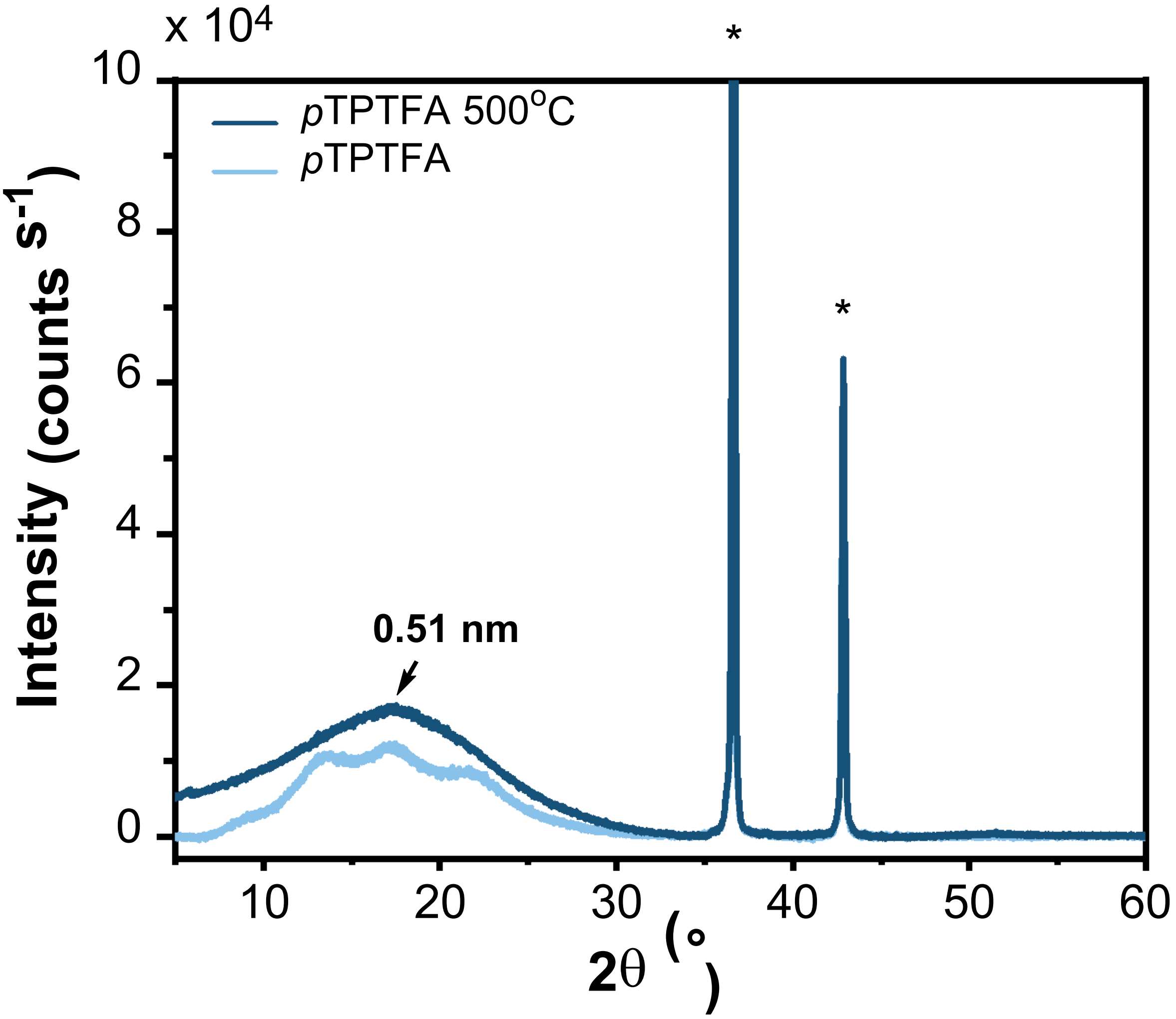

Supplementary Fig. 20. Wide-angle X-ray diffraction patterns of *p*TPTFA and *p*TPTFA 500 °C. The characteristic peak for the aluminum sample holder is highlighted with an asterisk.

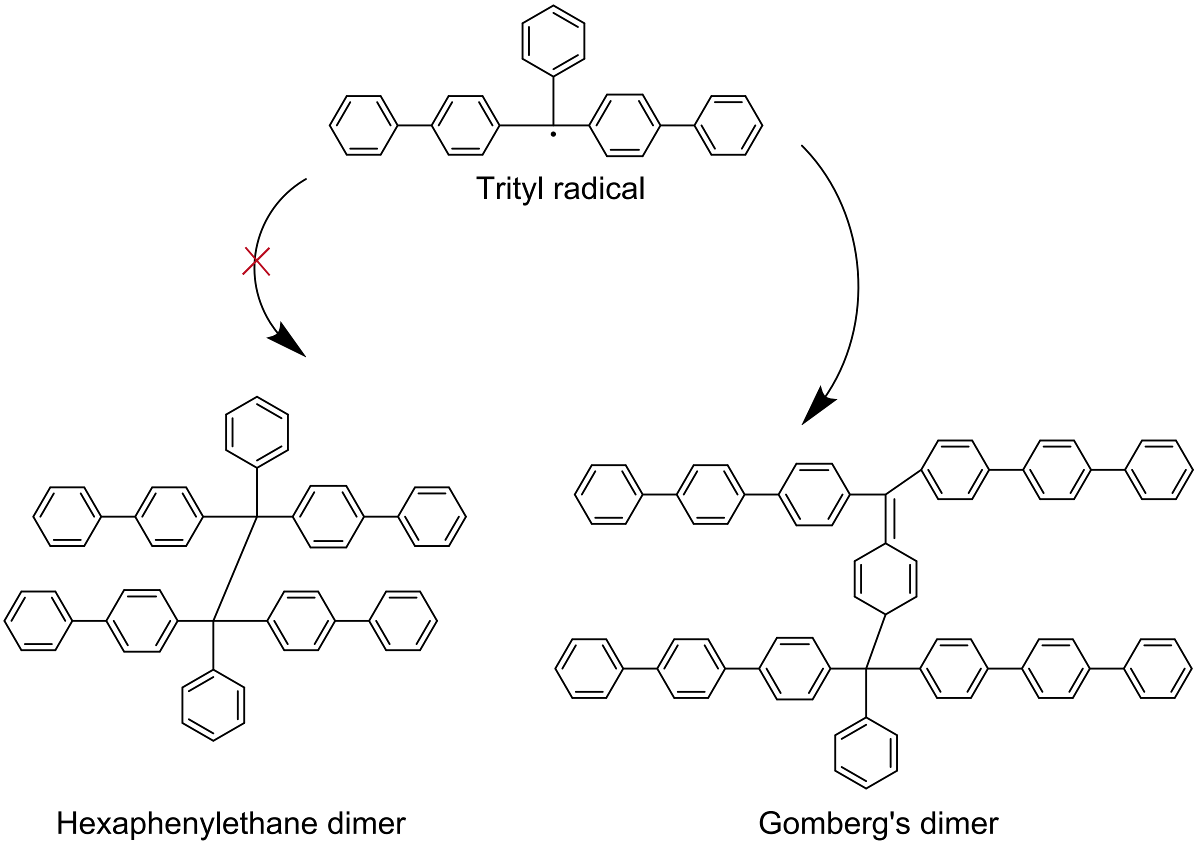

Supplementary Fig. 21. A proposed chemical structure of *p*TPTFA 500 °C.

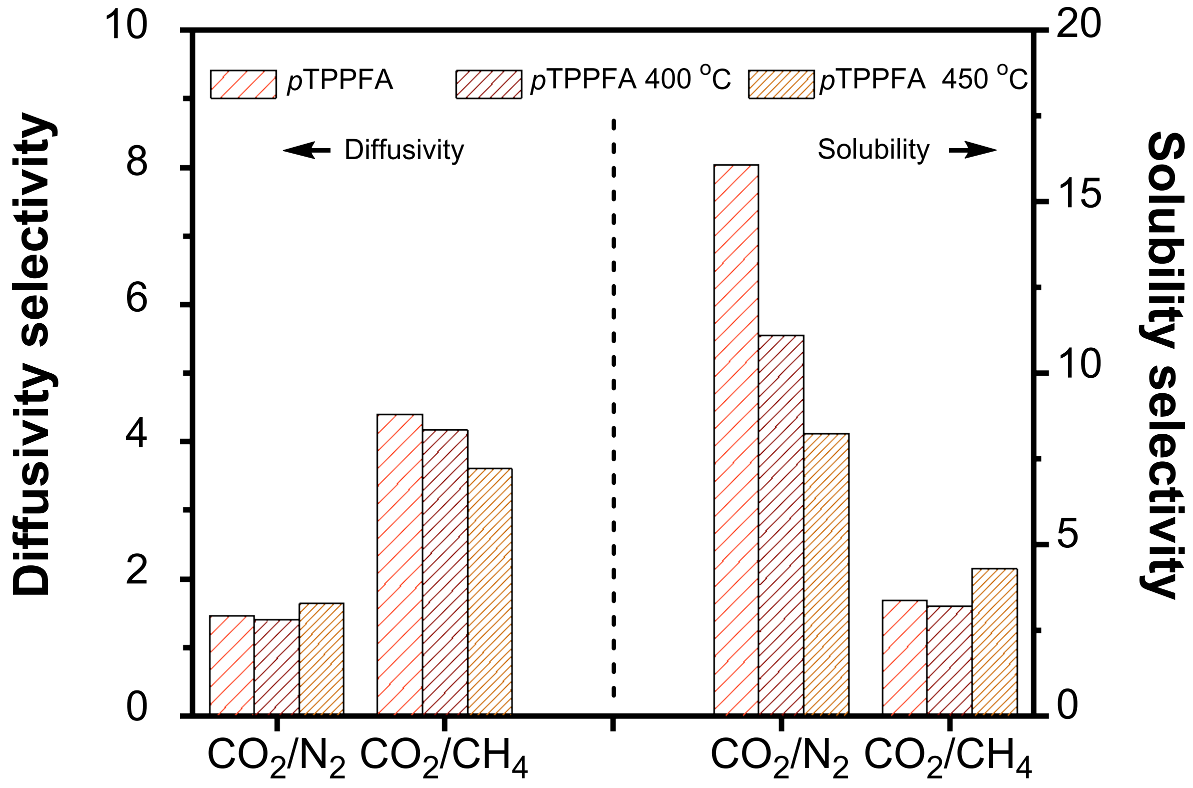

Supplementary Fig. 22. Diffusivity and solubility selectivity of CO_2/N_2 and CO_2/CH_4 for *p*TPPFA and its thermally treated derivatives at 1 bar and 35 °C.

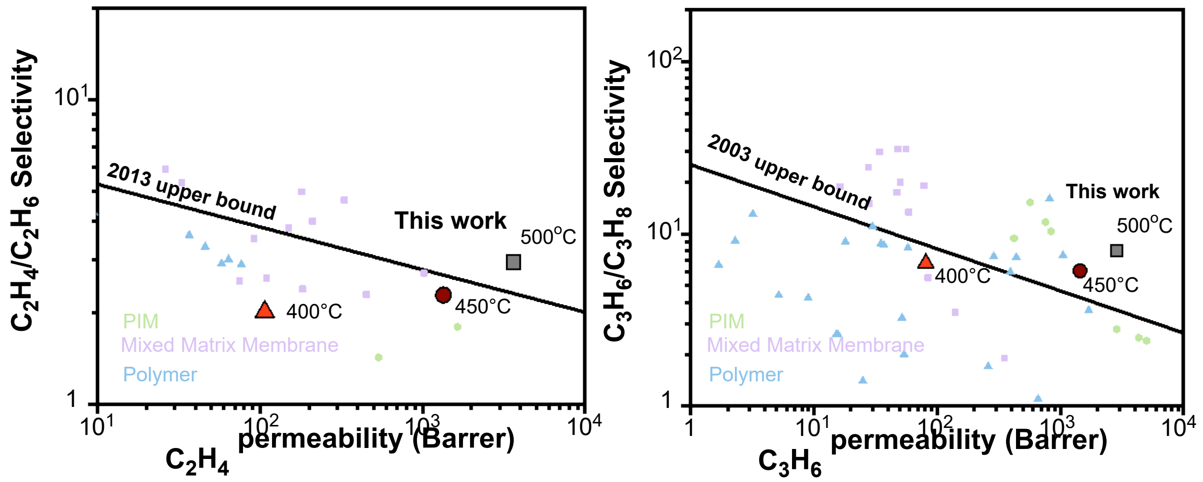

Supplementary Fig. 23. Comparisons of the single-gas separation performance of thermally treated *p*TPPFA for (a) C_2H_4/C_2H_6 and (b) C_3H_6/C_3H_8 measured at 2 bar and 35 °C.

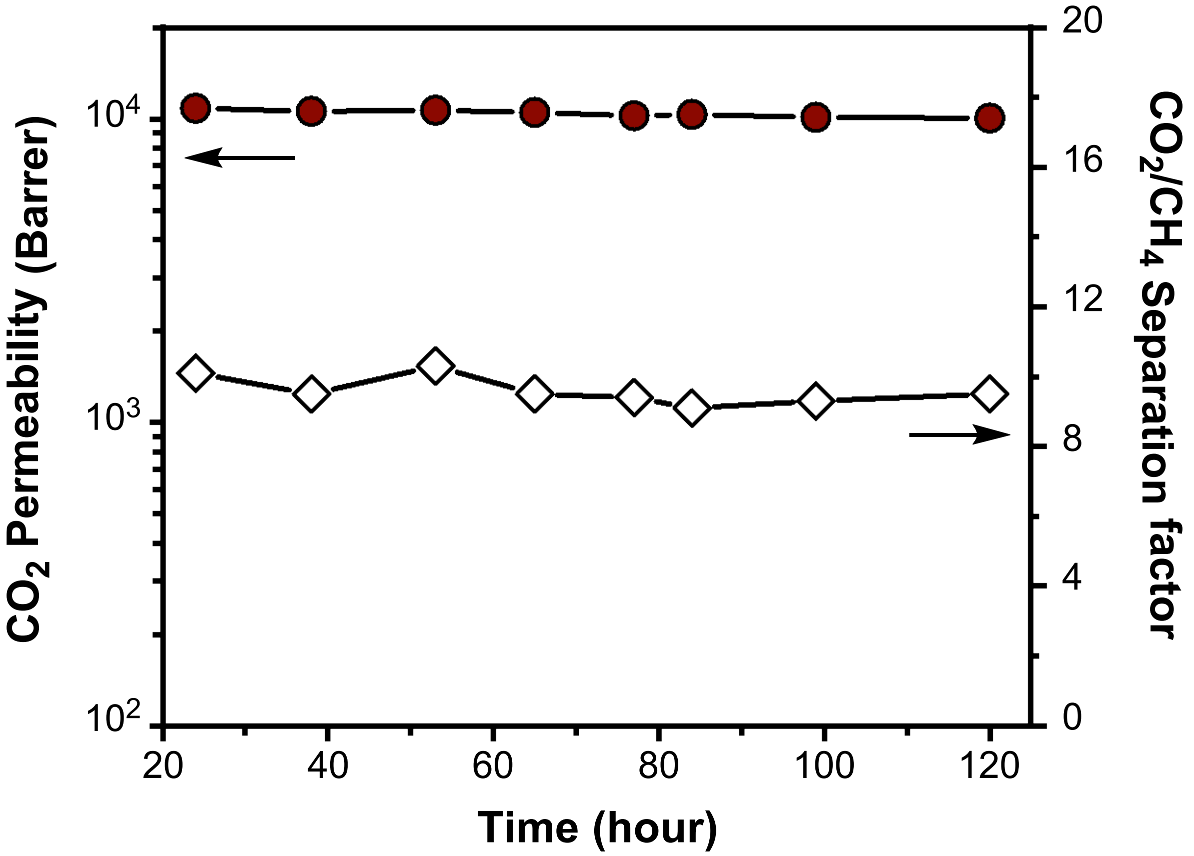

Supplementary Fig. 24. Long-term mixed-gas permeation performance of *p*TPPFA 450 °C over 96 hours of continuous operation.

The membrane was tested using a 50/50 mol% CO_2/CH_4 mixture at 2 bar and 35 °C. Both CO_2 and CH_4 permeabilities remained stable throughout the test, confirming excellent operational stability and resistance to physical aging.

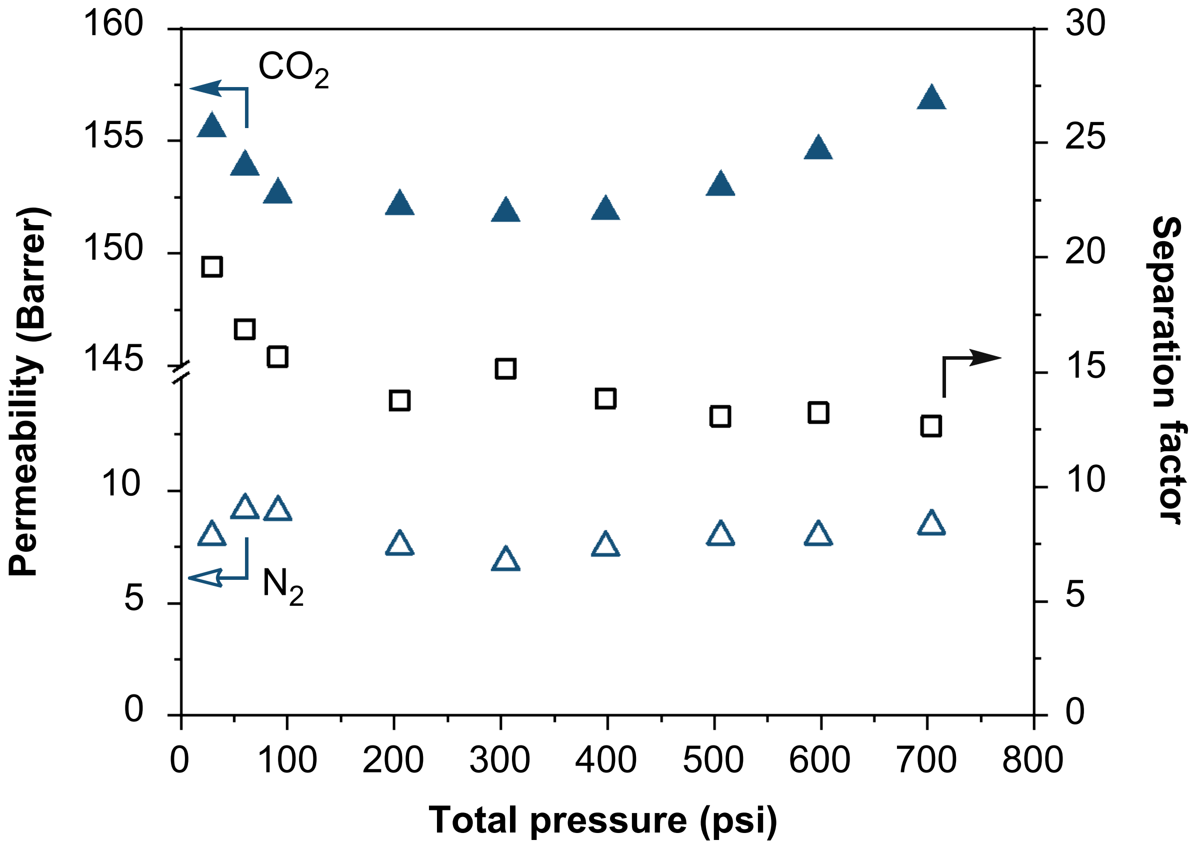

Supplementary Fig. 25. Equimolar CO_2/CH_4 mixed-gas separation performance as a function of total feed pressure for *p*TPPFA at 35 °C.

Note that the membrane without thermal treatment shows plasticization at pressures above 300 psi.

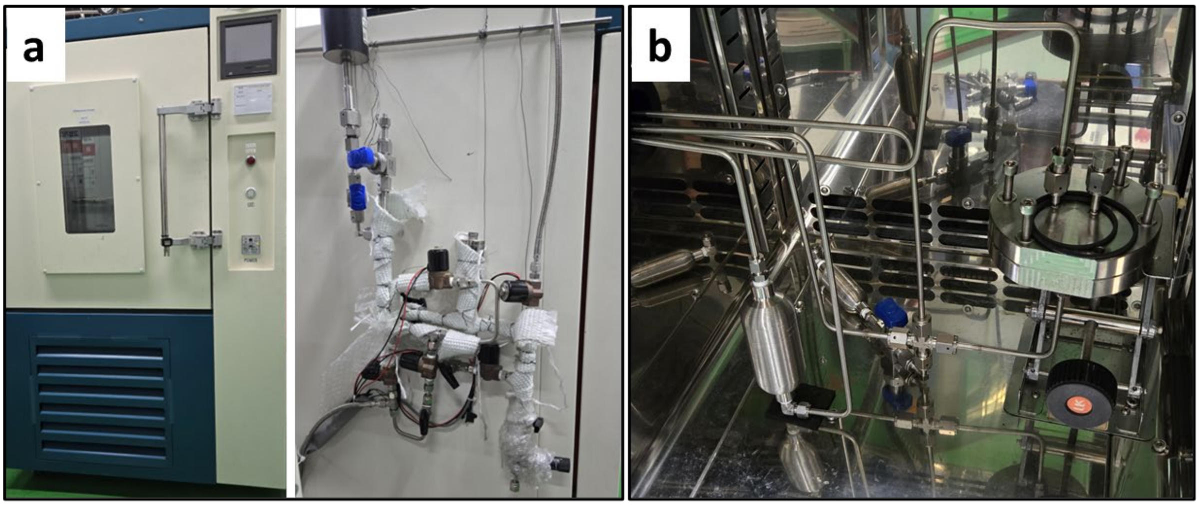

Supplementary Fig. 26. Photographs of (a) the exterior and (b) the interior of the in-house permeation system for sub-ambient temperature.

Supplementary Table 1. Molecular weight, glass transition temperature, density, fractional free volume, and *d*-spacing of *p*TPTFA and *p*TPPFA.

Polymer	M_n (g·mol^{-1})	M_w (g·mol^{-1})	T_g (℃)	ρ (g·cm^{-3})	FFV	*d*-spacing (Å)
*p*TPTFA	22,000	99,000	394	1.22	0.177	6.5
*p*TPPFA	35,000	61,000	335	1.42	0.181	6.8

Supplementary Table 2. Summary of mechanical properties of *p*TPPFA and *p*TPPFA 450°C membranes obtained from tensile stress-strain measurements.

Properties	Tensile strength (MPa)	Elongation strain at break (%)	Young's modulus (Gpa)
*p*TPPFA	60.2 ± 4.7	8.22 ± 0.62	1.09 ± 0.07
pTPPFA 450 °C (EMPM)	68.9 ± 2.1	2.39 ± 0.27	3.93 ± 0.46

Supplementary Table 3. BET surface area of EMPMs derived from thermally cross-linked *p*TPPFA and *p*TPTFA.

Properties	*p*TPTFA 500 °C	*p*TPPFA 450 °C	*p*TPPFA 500 °C*
BET surface area (m^2 g^{-1})	158	552	770
Micropore volume (>10 Å) (cm^3 g^{-1})	0.03	0.14	0.26
Total pore volume (cm^3 g^{-1})	0.08	0.23	0.30

*Note that *p*TPPFA 500 °C is a CMS membrane.

Supplementary Table 4. Dual-mode parameters of *p*TPPFA and EMPM (450 °C) for CO_2 adsorption isotherms.

Parameters	*p*TPPFA	EMPM (450 °C)*
$k_{D,CO2}$ $(cm^3(STP)\cdot cm^{-3}\cdot psi^{-1})$	0.134	0.106
$C'_{H,CO2}$ $(cm^3(STP)\cdot cm^{-3})$	23.1	58.8
b_{CO2} (psi^{-1})	0.030	0.0218

*Note that EMPM (450 °C) represents *p*TPPFA 450 °C hollow fiber membranes.

Supplementary Table 5. Partial immobilization model diffusion parameters for CO_2 adsorption isotherms.

Parameters	*p*TPPFA	EMPM (450 °C)
$D_{D,CO2}$ $(10^{-7}\ cm^2\cdot s^{-1})$	5.74	158
$D_{H,CO2}$ $(10^{-7} cm^2\cdot s^{-1})$	0.851	47.7
$F_{,CO2}$	0.148	0.302

Supplementary Table 6. Single-gas permeation results of freshly prepared asymmetric *p*TPPFA EMP hollow fiber membranes at 1 bar.

Testing Temperature (°C)	Gas	Permeability (GPU)[a]	Selectivity[b]	
			CO_2/N_2	CO_2/CH_4
20	CO_2	4,848	10.4	10.9
	N_2	464		
	CH_4	444		
-20	CO_2	2,174	30.6	55.9
	N_2	71.0		
	CH_4	38.9		

[a] 1 GPU = 10^{-6} cm^3 (STP) cm^{-2} s^{-1} $cmHg^{-1}$. [b] Permselectivity ($\alpha_{A/B}$) = ideal P_A/P_B.

Supplementary Table 7. Long-term single-gas permeation results of asymmetric *p*TPPFA EMP hollow fiber membranes at 1 bar and sub-ambient temperature (-20 °C). The testing module was stored under a vacuum at -20 °C.

Aging time	Gas	Permeability (GPU)[a]	Selectivity[b]	
			CO_2/N_2	CO_2/CH_4
2 days	CO_2	2,048	30.6	55.9
	N_2	71.0		
	CH_4	38.9		
17 days	CO_2	1,708	35.6	64.1
	N_2	48		
	CH_4	26.6		

[a] 1 GPU = 10^{-6} cm^3 (STP) cm^{-2} s^{-1} $cmHg^{-1}$. [b] Permselectivity ($\alpha_{A/B}$) = ideal P_A/P_B.

Supplementary Table 8. Mixed-gas permeation results of asymmetric *p*TPPFA EMP hollow fiber membranes at 2 bar and sub-ambient temperature (-20 °C).

Aging time	Gas	Permeability (GPU)[a]	Separation Factor[b]	
			CO_2/N_2	CO_2/CH_4
18 days	CO_2	1,654	29.6	55.8
	N_2	55.9		
	CH_4	29.6		

[a] 1 GPU = 10^{-6} cm^3 (STP) cm^{-2} s^{-1} $cmHg^{-1}$. [b] Separation factor ($\alpha_{A/B}$) = $(y_A/y_B)_{permeate}/(x_A/x_B)_{feed}$.

Supplementary Table 9. Comparison of CO_2/N_2, CO_2/CH_4 separation performance between polymeric membranes and thermally cross-linked membranes.

Sample	Testing condition	CO_2 Permeability (Barrer)	CO_2/N_2 Selectivity	CO_2/CH_4 Selectivity	References
This work					
*p*TPTFA	35 °C, 1 bar	184	24	15	-
*p*TPTFA 500 °C (EMPM)		4,632	17	10	
*p*TPPFA		280	16.5	14.7	
*p*TPPFA 400 °C		919	15.6	13.3	
*p*TPPFA 450 °C (EMPM)	35 °C, 1 bar	12,162 ± 979	13.2 ± 0.102	12.0 ± 0.258	
	20 °C, 1 bar	12,788	16.9	17.9	
	0 °C, 1 bar	13,276	25.3	34	
	-20 °C, 1 bar	10,324	46.4	61.3	
Polymeric Membranes					
PIM-1	25 °C, 14.7 psi	2,300	25	18.4	3
PIM-1	10 °C, 29 psi	3,159	29.3	22.1	4
	0 °C, 29 psi	2,678	35.7	27.9	
	-10 °C, 29 psi	2,390	41.9	36.2	
	-20 °C, 29 psi	1,946	55.6	47.5	
	-30 °C, 29 psi	1,380	81.2	65.7	
DFTTB Ladder polymer	35 °C, 2 bar	3,146	28.9	21.8	5
IFTTB Ladder polymer	35 °C, 2 bar	3,901	21	14.1	
Spirobifluorene-based PIM	25 °C, 14.7 psi	13,900	17.6	12.6	6
Tröger's base (TB)-based PIM	35 °C, 14.7 psi	4353	18	12	7
PIM-TMN-Trip	25 °C, 14.7 psi	52,800	14.9	7.3	8
PIM-HMI-Trip		44,200	17.3	9.1	
PIM-DTFM-BTrip		42,600	14.2	9.8	
PIM-TFM-BTrip		33,700	18.4	14.8	
CANAL-Me-Me2F	25 °C, 14.7 psi	5,400	14.6	9.4	9
CANAL-Me-S5F		4,000	18.2	13	
CANAL-ME-S6F		2,900	15.5	10	
CANAL-Me-DHP		3,400	14.5	8.5	

PIM-TMN-SBI	25 °C, 14.7 psi	17,500	16.2	8.3	10
PIM-TMN-Trip-TB		6,060	15.3	24.6	
Thermal cross-linked membrane					
6FDA-DAM:DABA(3:2) Before heat treatment	35 °C, 100 psi	161	21.4	34	11
6FDA-DAM:DABA(3:2) After heat treatment for 370 °C, 1hour		485	19.3	27	
75%-Br-6FDA-DAM-DABA(3:2) Before heat treatment	35 °C, 2 bar	88.3	26.8	30.4	12
75%-X-Br-6FDA-DAM-DABA(3:2) After heat treatment for 360 °C		3,177.3	14.6	14.9	
PI-Br Before heat treatment	35 °C, 2 bar	62.7	28.5	69.7	13
PI-Br-450 After heat treatment for 450 °C		274	22.8	49.1	
6FDA-Durene:DABA (9:1)-g-CD Before heat treatment	35 °C, 20 atm	56	21.5	31	14
6FDA-Durene:DABA (9:1)-g-CD After heat treatment for 425 °C		4,016	10.6	16	
6FDA-Durene:DABA (9:1)-g-CD After heat treatment for 450 °C		8,000	15.2	17	
Thermally Rearranged (TR) polymer					
PIOFG-1 (Before TR)	35 °C, 10 atm	10	25	125	15
TR-1-450		2,045	26	44	

References

1. Bondi, A. van der Waals Volumes and Radii. *J. Phys. Chem.* **68**, 441–451 (1964).
2. Paul, D. R. & Koros, W. J. Effect of partially immobilizing sorption on permeability and the diffusion time lag. *J. Polym. Sci. Polym. Phys. Ed.* **14**, 675–685 (1976).
3. Budd, P. M. *et al.* Gas separation membranes from polymers of intrinsic microporosity. *J. Membr. Sci.* **251**, 263–269 (2005).
4. Ji, W. *et al.* Remarkably enhanced gas separation properties of PIM-1 at sub-ambient temperatures. *J. Membr. Sci.* **623**, 119091 (2021).
5. Ma, X. *et al.* Unprecedented gas separation performance of a difluoro-functionalized triptycene-based ladder PIM membrane at low temperature. *J. Mater. Chem. A* **9**, 5404–5414 (2021).
6. Bezzu, C. G. *et al.* A Spirobifluorene-Based Polymer of Intrinsic Microporosity with Improved Performance for Gas Separation. *Adv. Mater.* **24**, 5930–5933 (2012).
7. Gong Wang, Z., Liu, X., Wang, D. & Jin, J. Tröger's base-based copolymers with intrinsic microporosity for CO 2 separation and effect of Tröger's base on separation performance. *Polym. Chem.* **5**, 2793–2800 (2014).
8. Comesaña-Gándara, B. *et al.* Redefining the Robeson upper bounds for CO2/CH4 and CO 2/N2 separations using a series of ultrapermeable benzotriptycene-based polymers of intrinsic microporosity. *Energy Environ. Sci.* **12**, 2733–2740 (2019).
9. Lai, H. W. H. *et al.* Hydrocarbon ladder polymers with ultrahigh permselectivity for membrane gas separations. *Science* **375**, 1390–1392 (2022).
10. Rose, I. *et al.* Polymer ultrapermeability from the inefficient packing of 2D chains. *Nat. Mater.* **16**, 932–937 (2017).
11. Qiu, W. *et al.* Sub-Tg Cross-Linking of a Polyimide Membrane for Enhanced CO2 Plasticization Resistance for Natural Gas Separation. *Macromolecules* **44**, 6046–6056 (2011).
12. An, H. *et al.* Bromination/debromination-induced thermal crosslinking of 6FDA-Durene for aggressive gas separations. *J. Membr. Sci.* **545**, 358–366 (2018).
13. Sun, L. *et al.* The influence of debromination and TR on the microstructure and properties of CMSMs. *Sep. Purif. Technol.* **352**, 128167 (2025).
14. Xiao, Y. & Chung, T.-S. Grafting thermally labile molecules on cross-linkable polyimide to design membrane materials for natural gas purification and CO2 capture. *Energy Environ. Sci.* **4**, 201–208 (2010).
15. Park, H. B. *et al.* Polymers with Cavities Tuned for Fast Selective Transport of Small Molecules and Ions. *Science* **318**, 254–258 (2007).
