# Supplementary material for: Extrinsically microporous polymer membranes derived from thermally cross-linked perfluorinated aryl-ether-free polymers for gas separation
Source: Nat Commun. 2025 Aug 5;16:7143. doi: 10.1038/s41467-025-62372-y (PMC12325600; doi:10.1038/s41467-025-62372-y)
Supplement: Supplementary file 3 — Description of Additional Supplementary Files [file 41467_2025_62372_MOESM3_ESM.pdf]

Description of Additional Supplementary Files

Supplementary Movie 1. Flexibility test for carbon molecular sieve membrane

Supplementary Movie 2. Flexibility test for extrinsically microporous polymeric membrane
